# Screening of cognitive performance in kidney transplant recipients: a mini review

**DOI:** 10.3389/fneph.2023.1238501

**Published:** 2023-09-14

**Authors:** Simeon Schietzel, Reto W. Kressig, Uyen Huynh-Do

**Affiliations:** ^1^ Division of Nephrology and Hypertension, Inselspital, Bern University Hospital, University of Bern, Bern, Switzerland; ^2^ University Department of Geriatric Medicine FELIX PLATTER, and University of Basel, Basel, Switzerland

**Keywords:** transplantation, cognition, screening, kidney, dementia

## Abstract

**Why should we screen?:**

The prevalence of cognitive impairment in kidney transplant recipients (KTRs) is up to 58%. The 10-year graft loss and mortality rates are above 30% and 50%, respectively, and executive malfunctioning increases disadvantageous outcomes.

**What causes cognitive impairment in KTRs?:**

Strong risk factors are older age and chronic kidney disease. However, causes are multifactorial and include cardiovascular, cerebrovascular, neurodegenerative, inflammatory, uremic, psychiatric, and lifestyle-related susceptibilities.

**How should we screen?:**

KTR-specific validated instruments or strategies do not exist. The central element should be a multidomain cognitive screening test that is sensitive to mild cognitive impairment, corrects for age and education, and includes executive functions testing. Cognitive trajectories, effects on everyday life and psychiatric comorbidities should be assessed by integrating the perspectives of both patients and knowledgeable informants.

**When should we screen?:**

Screening should not be postponed if there is suspicion of impaired cognition. Different time points after transplantation tend to have their own characteristics.

**Who should conduct the screening?:**

Screening should not be limited to specialists. It can be carried out by any healthcare professional who has received a limited amount of training.

**What are the benefits of screening?:**

Screening does not provide a diagnosis. However, suggestive results change care in multiple ways. Goals are: Initiation of professional dementia work-up, securing of adherence, anticipation of potential complications (delirium, falls, frailty, functional impairment, malnutrition, etc.), mitigation of behavioral disorders, adjustment of diagnostic and therapeutic “load”, reduction of caregiver burden and meeting of changing needs. We summarize data on the prevalence, risk factors and sequelae of cognitive impairment in KTRs. We also discuss the requirements for appropriate screening strategies and provide guiding principles regarding appropriate and safe care.

## Introduction

Cognitive impairment in kidney transplant recipients (KTRs) is a challenge for both patients and caregivers. Existing studies suggest a prevalence of up to 58% (1) and a 10-year incidence of up to 17% (2). Cognitive impairment in KTRs significantly impacts prognosis: the 10-year graft loss and mortality rates for these KTRs are > 30% and > 50% higher, respectively, than those of KTRs without impaired cognition (2). Pharmacological treatment options are limited. However, in cases of impaired cognition, the care of KTRs needs to be adjusted in multiple ways. Data regarding KTR-specific screening strategies and effective care, which can reduce the risks and improve the prognosis of KTRs with cognitive impairment, are scarce.

### How common is cognitive impairment in the kidney transplant patient population?

The existing literature on the frequency of cognitive impairment in KTRs is scarce and is summarized in **Table 1**. In a study conducted by the Johns Hopkins Hospital, Baltimore, MD, USA, and the University of Michigan Medical Center, Ann Arbor, MI, USA, which applied the Modified Mini Mental State Exam (3MS (10), with a cutoff value < 80), the prevalence of cognitive impairment in 92 KTRs aged above 65 years was found to be 6.5% (7). Seventy-three percent were educated to above high school level, the median Charlson Comorbidity Index score was low [2; interquartile range (IQR) 0.2, 3], the median dialysis vintage was 1.7 (IQR 0.4, 4) years, and testing was carried out 1 year after transplantation.

**Table 1:** Cognition screening tests in kidney transplant patients.

Test	Domains screened Test characteristics	Author, year and study population	Number and mean age (years) (SD)	Education	Testing after KT	Results	Comments
Global cognition screening tests—instruments for an initial and general screening of cognitive functions							
**MoCA**	Memory, executive functioning, attention, language, visuospatial, and orientation. Time required: 10 minutes. Maximum value 30 points; and cutoff value < 26 points. Validation in KTRs: no. Correction for age: no. Correction for education: yes (plus 1 point if education ≤ 12 years).	Gupta 2017 (1), USA, 2015–2016, eGFR 52 (21), and dialysis vintage 2.3 (1.2)	*n* = 226 age 54 (13)	58% college graduates	3.4 (4.1) years	Cognitive impairment (<26 points): 58%	Very suitable for KTRs. Substantial comparative data from non-KTR populations available
		Gupta 2022 (3), Italy, 2015–2019, 27%, pre-emptive KT 68%, and dialysis vintage 1.8 (2.6)	*n* = 108 age 47 (14)	20% high school completed; 20% college completed	0.9 (0.9) years	Mean score: 26.4 (2.8)	
**DemTect**	Memory, language, executive functioning, attention. Time required: 8 minutes. Maximum value 18 points; and cutoff value < 13 points. Validation in RTR: no. Correction for age: yes. Correction for education: yes (plus 1 point if education ≤ 11 years).	Nöhre 2019 (4), Germany, eGFR 45.8 (18.4), HADS score: 4.35 (4), and dialysis vintage 5.0 (4.1)	*n* = 583, age 52.1 (14.3)	27% ≥ 12 years	5.5 (5.7) years	Cognitive impairment (< 13 points): 15.6%. Conversion into MoCA scores: mean 27.2 (SD 3.2), cognitive impairment (< 26 points) 22.3%	Very suitable for KTRs. Comparative data from non-KTR populations available
**MMSE**	Memory, language, visuospatial, orientation, attention, understanding/following instructions. Time required: 5 minutes–10 minutes. Maximum value 30 points; cutoff value < 24 points. Validation in RTR: no. Correction for age or education: no.	Harciarek 2009 (5), Poland, 2005–2007, creatinine 740 (254), Hb 118(13), and dialysis vintage 2.8 (2.9)	*n* = 22 age 47.1 (11.4)	12.3 (2.5) years	30 (25) days	Mean score 28.7 (1.2)	Best-known global cognition screening test. Suitable for KTRs if executive function testing is also carried out (e.g., clock drawing, TMT-B, verbal fluency). Substantial comparative data from non-KTR populations available
		Gupta 2016 (6), USA, and dialysis vintage 2.23 (1.85)	*n* = 11 age 56.5 (10.7)	High school, *n* = 3; 4-year college, *n *= 4	3 months	Mean score 28.2 (2.6)	
**3MS**	Memory, language, visuospatial, orientation, attention, understanding/following instructions, executive functions. Time required: 8 minutes–15 minutes. Maximum value, 100 points; cutoff value <78 points. Validation in RTR: no. Correction for age or education: no.	Chu 2021 (7), USA, 2009–2019, Charlson Comorbidity Index score 2 (IQR 0.2, 3), and dialysis vintage 1.7 (IQR 0.4, 4)	*n* = 405 age 18 to > 65	73% above high school	1 year	Cognitive impairment (< 80 points): 1.7% of those aged 18–34 years; 3.4% of those aged 35–49 years; 4.3% of those aged 50–64 years; 6.5% of those aged > 64 years	Important extension of the MMSE incorporating executive functions testing. Very suitable global cognition screening test for KTRs. Comparative data from non-KTR populations available
**RBANS**	Memory, attention, language, visuospatial. 25 minutes. Maximum value, 160 points, cutoff value ≤ 85 points. Validation in RTR: no. Correction for age or education: no.	Binari 2022 (8), USA, 2017–2019, and dialysis vintage 2.5 (2.0)	*n* = 31 age 44.9 (12.1)	High school, college, post graduate degree: 25%, 19%, and 16%,	3 months and 12 months	3 months: mean 87.5 (14.3) 12 months: mean 85.1 (14.2)	Sensitive screening test. Enables longitudinal evaluation. Suitable for KTRs if executive function testing is also carried out (e.g., clock drawing, TMT-B, verbal fluency). Comparative data from non-KTR populations available
Single cognitive domains screening tests—for an additional evaluation							
**TMT-A**	Visual search, attention, processing speed, motor speed. Measured in seconds	Griva 2006 (9), UK 1998–2000, creatinine 135.4 (8.3), and Hb 131 (17)	*n* = 28 age 44 (12)	Age left school: 18.7 (6.4) years	6 months	Mean 32.5 (17.5), maximum 68.8, impaired 17.9%	Popular and recommendable test for its domains. Focus on speed. Short and easy to conduct
		Harciarek 2009 (5), Poland, 2005–2007, creatinine 740 (254), and Hb 118 (13), and dialysis vintage 2.8 (2.9)	*n* = 22 age 47.1 (11.4)	12.3 (2.5) years	30 (25) days	Mean 36.1 (7.7)	
		Binari 2022 (8), USA, 2017–2019, and dialysis vintage 2.5 (2.0)	*n* = 31 age 44.9 (12.1)	High school, college, postgraduate degree: 25%, 19%, and 16%,	3 months and 12 months	3 months: mean 44.7(11) 12 months: mean 45.6 (11.9)	
**TMT-B**	Executive function test, especially organized visual search, planning, attention, processing speed, motor speed, set shifting, inhibition, cognitive flexibility, and divided attention. Measured in seconds	Griva 2006 (9), UK, 1998–2000, creatinine 135.4 (8.3), and Hb 131 (17)	*n* = 28 age 44 (12)	Age left school: 18.7 (6.4) years	6 months	Mean 77.2 (41.8), range 182.9, impaired 14.3%	Very popular and highly recommendable test for its domains, especially executive functions. Short and easy to conduct
		Harciarek 2009 (5), Poland, 2005–2007, creatinine 740 (254), Hb 118(13), and dialysis vintage 2.8 (2.9)	*n* = 22 age 47.1 (11.4)	12.3 (2.5) years	30 (25) days	Mean 93.18 (41.33)	
		Binari 2022 (8), USA, 2017–2019, and dialysis vintage 2.5 (2.0)	*n* = 31 age 44.9 (12.1)	High school, college, postgraduate degree: 25%, 19%, and 16%,	3 months and 12 months	3 months: mean 48.4 (12.2) 12 months: mean 46.6 (11.3)	
**RAVLT**	Memory. Provides different measures of learning and memory. Here, amount of total words recalled (from five word list runs)	Griva 2006 (9), UK, 1998–2000, creatinine 135.4 (8.3), and Hb 131 (17)	*n* = 28 age 44 (12)	Age left school: 18.7 (6.4) years	6 months	Mean 53.21 (9.2), range 38, impaired 14.3%	Popular and in detail evaluation of learning and memory functions. Recommendable for advanced screening
		Harciarek 2009 (5), Poland, 2005–2007, creatinine 740 (254), Hb 118(13), and dialysis vintage 2.8 (2.9)	*n* = 22 age 47.1 (11.4)	12.3 (2.5) years	30 (25) days	Mean 49.32 (7.4)	
**Verbal fluency** Letter	Language, executive function, and frontal search process. Measured in words per minute. For Polish adaption letter “K” instead of letter “F” was used	Harciarek 2009 (5), Poland, 2005–2007, creatinine 740 (254), Hb 118(13), and dialysis vintage 2.8 (2.9)	*n *= 22 age 47.1 (11.4)	12.3 (2.5) years	30 (25.2) days	13.64 (4.1)	Popular and highly recommendable test for its domains. Short and easy to conduct
**Verbal fluency** Category	Language, executive function, and frontal search process. Measured in words per minute. “Animals” were applied as category	Harciarek 2009 (5), Poland, 2005–2007, creatinine 740 (254), Hb 118(13), and dialysis vintage 2.8 (2.9)	*n* = 22 age 47.1 (11.4)	12.3 (2.5) years	30 (25) days	18.77 (4.4)	Popular and highly recommendable test for its domains. Short and easy to conduct
**Digit Span** Forward	Attention, short-term memory, and working memory. Measured in number of digits remembered	Harciarek 2009 (5), Poland, 2005–2007, creatinine 740 (254), Hb 118(13), and dialysis vintage 2.8 (2.9)	*n* = 22 age 47.1 (11.4)	12.3 (2.5) years	30 (25) days	5.91 (1.7)	Popular and recommendable test for its domains. Short and easy to conduct
**Digit Span** Backward	Attention, short-term memory, working memory, and executive function. Measured in the number of digits remembered	Harciarek 2009 (5), Poland, 2005–2007, creatinine 740 (254), Hb 118(13), and dialysis vintage 2.8 (2.9)	*n* = 22 age 47.1 (11.4)	12.3 (2.5) years	30 (25) days	5.68 (1.9)	Popular and highly recommendable. Very valuable variant of digit span forward being more difficult and requiring executive functions
**DSST**	Attention, processing speed, motor speed, working memory, learning, and executive functions. Measured in the number of symbols per 90 s	Harciarek 2009 (5), Poland, 2005–2007, creatinine 740 (254), Hb 118(13), and dialysis vintage 2.8 (2.9)	*n *= 22 age 47.1 (11.4)	12.3 (2.5) years	30 (25) days	Mean 42.5 (9.6)	Popular test for its domains. Recommendable for advanced screening
**SDMT**	Inverse of DSST. Attention, processing speed, motor speed, working memory, learning, and executive functions. Measured in no. of digits per 90 s. Written (w) and oral (o)	Griva 2006 (9), UK, 1998–2000, creatinine 135.4 (8.3), and Hb 131 (17)	*n* = 28 age 44 (12)	Age left school: 18.7 (6.4) years	6 months	w: mean 53.3 (13.7), range 51, and impaired 17.9% o: mean 59.2 (15.2), range 56, and impaired 17.9%	Popular test for its domains. Recommendable for advanced screening
**BVRT**	Drawing visual designs by heart after 10 s of presentation. Visual memory, visual perception, and visual construction. Measured in the number of correct reproductions (c) and number of reproduction errors (e)	Griva 2006 (9), UK, 1998–2000, creatinine 135.4 (8.3), and Hb 131 (17)	*n* = 28 age 44 (12)	Age left school: 18.7 (6.4) years	6 months	c: mean 7.1 (2), range 6, and impaired 3.6% e: mean 4.1 (3.3), range 10, and impaired 10.7%	Popular test for its domains. Recommendable for advanced screening
**GP**	Twenty-five pegs with a key along one side must be inserted (one line after the next, in correct rotation) into 25 holes with randomly positioned slots. Visual–motor coordination, fine motor skills, and speed. Measured in seconds for dominant (d) and non-dominant (non-d) hand	Griva 2006 (9), UK, 1998–2000, creatinine 135.4 (8.3), and Hb 131 (17)	*n* = 28 age 44 (12)	Age left school: 18.7 (6.4) years	6 months	d: mean 75.3 (22.6), range 103.7, and impaired 18.5% Non-d: mean 86.6 (27.9), range 112.1, and impaired 10.7%	Popular test for its domains. Recommendable for advanced screening

KT, kidney transplantation; MoCA, Montreal Cognitive Assessment; eGFR, estimated glomerular filtration rate, given as mean (SD) in ml/min/1.73 m^2^; education is given in mean (SD) years; dialysis vintage is given in mean (SD) years; DemTect, Demenz Detection Test; HADS score, Hospital Anxiety and Depression Scale score, given in mean (SD) points; MMSE, Mini Mental State Examination; creatinine values are given as mean (SD) in µmol/L; Hb, hemoglobin given as mean (SD) in g/L; 3MS, Modified Mini Mental Test; RBANS, Repeatable Battery for the Assessment of Neuropsychological status; TMT-A or -B, Trail Making Test A or B; RAVLT, Rey Auditory Visual Learning Test; DSST, Digit Symbol Substitution Test; SDMT, Symbol Digit Modalities Test; BVRT, Benton Visual Retention Test; GP, Grooved Pegboard Test.

In a German cohort of 583 KTRs, with a mean age of 52.1 (SD 14.3) years, the prevalence of cognitive impairment was found to be higher (15.6%) using the DemTect cognition screening test (11) (with a cutoff value < 13) (4). Twenty-seven percent had undergone education for ≥ 12 years, the mean estimated glomerular filtration rate (eGFR) was 45.8 (SD 18.4) mL/min/1.73 m^2^, the dialysis vintage was 5.0 (4.1) years, and testing was carried out 5.5 (SD 5.7) years after transplantation.

In a study of 226 KTRs with a mean age of 54 (SD 13.4) years at the University of Kansas Kidney Transplant Clinic, prevalence of cognitive impairment was even 58% using the Montreal Cognitive Assessment (MoCA) test (12) (with a cutoff value < 26) (1). Fifty-eight percent were college graduates, the dialysis vintage was 2.3 (2.1) years, the mean eGFR was 52 (SD 21) mL/min/1.73 m^2^, and testing was carried out 3.4 (4.1) years after transplantation.

McAdams-DeMarco et al. investigated data from 40,918 US KTRs and found a 10-year dementia risk of 5.1% for recipients aged between 55 years and 60 years, stepwise rising to 17% for participants aged ≥75 years (2). Dementia was defined using the reported diagnoses within the *International Classification of Diseases* (ICD) system. Incidence rates were calculated linking KTR data to Medicare claims through the US Renal Data system.

The prevalence of cognitive impairment in the general population appears to be lower. European meta-analysis data investigating *Diagnostic and Statistical Manual of Mental Disorders*, Fourth Edition (DSM-IV)-based dementia diagnoses of 18,263 participants, aged 65 years to > 90 years, found an age- and sex-standardized prevalence rate of 7.1% (13). However, the comparability among studies is very limited.

### Which patients are particularly at risk?

The strongest and most intuitive risk factor for cognitive impairment in KTRs is age. Chu et al. found the prevalence of cognitive impairment of KTRs to steadily increase from 1.7% (18–34 years) to 6.5% (≥ 65 years) (7). McAdams-DeMarco et al. found an adjusted HR for dementia onset of 1.5 (95% CI 1.46 to 1.56) per every five-year increase in age (2). Gupta et al. found an adjusted odds ratio (OR) of 1.33 for cognitive impairment per every 10-year increase in age (1). Other factors that have been independently associated with cognitive impairment in KTRs are lower education level (2, 4, 14), lower eGFR (4), diabetes (2), more years on dialysis (2), hypertension (4), frailty (15), depressive symptoms, and less capability regarding the activities of daily living (14) (**Table 2**). However, the results among the studies are far from consistent.

**Table 2:** Factors independently associated with cognitive impairment in kidney transplant recipients.

Factor	Author and year	Results, effect size
Older age*	Chu et al., 2021 (7)	Age and prevalence (%) of cognitive impairment (3MS score < 80): 18–34 years: 1.7% 35–49 years: 3.4% 50–64 years: 4.3% > 64 years: 6.5%
	McAdams-DeMarco et al., 2017 (2)	HR dementia (ICD) onset per 5 years: 1.5 (95% CI 1.46 to 1.56) 10-year risk of dementia: 55–60 years: 5.1% 60–65 years: 7.2% 65–70 years: 11.0% 70–75 years: 15.6% ≥ 75 years: 17.0%
	Gupta et al., 2017 (1)	OR cognitive impairment (MoCA score < 26) per every 10-year increase in age: 1.33 (95% CI 1.06 to 1.69)
Lower education level*	McAdams-DeMarco et al., 2017 (2)	HR dementia, no education vs. associate or bachelor’s degree: 1.66 (95% CI 1.10 to 2.49)
	Nöhre et al., 2019 (4)	OR cognitive impairment (DemTect <13) if educated in school for < 12 years: 2.4 (95% CI 1.3 to 4.7)
	Thind et al., 2022 (14)	Mean (range) years of education for cognitive impairment vs. no impairment: 17.4 (0 to 36) vs. 19.2 (12 to 40); *p* = 0.021
Diabetes*	McAdams-DeMarco et al., 2017 (2)	HR dementia: 1.64 (95% CI 1.51 to 1.78)
Dialysis Vintage*	McAdams-DeMarco et al., 2017 (2)	HR dementia per 5 years: 1.09 (95% CI 1.02 to 1.16)
**Lower kidney function**	Nöhre et al., 2019 (4)	OR cognitive impairment (DemTect < 13) if higher eGFR: 0.98 (95% CI 0.965 to 0.995) mL/min/1.73 m^2^
Donor type*	McAdams-DeMarco et al., 2017 (2)	HR dementia, living vs. deceased donor (SCD): 1.13 (95% CI 1.01 to 1.27). However, living donor vs. ECD or vs. DCD non-significant.
Hypertension*	Nöhre et al., 2019 (4)	OR cognitive impairment (DemTect < 13) if hypertensive: 2.64 (95% CI 1.02 to 6.88)
Frailty*	Chu et al., 2019 (15)	Percent cognitive impairment (3MS < 80) for frail vs. non-frail: 11% vs. 6.6%; *p* < 0.05
**Depressive symptoms**	Thind et al., 2022 (14)	Percent depressive symptoms for cognitive impairment vs. no impairment: 50.8% vs. 37.6%; *p* = 0.034
**Reduced autonomy**	Thind et al., 2022 (14)	Mean (range) Nottingham ADL score for cognitive impairment vs. no impairment: 14.4 (4 to 21) vs. 17 (2 to 22); *p* = 0.0016

3MS, Modified Mini Mental Test; ICD, International Classification of Diseases and Related Health Problems; MoCA, Montreal Cognitive Assessment; eGFR, estimated glomerular filtration rate; ADL, activities of daily living; SCD, standard criteria; ECD, extended criteria donor; DCD, donation after cardiac death.

*Other studies did not find such an association.

### Why do kidney transplant recipients suffer from cognitive impairment?

Chronic kidney disease (CKD) itself has been identified to be a strong independent risk factor for cognitive impairment. Undoubtedly, KTRs remain CKD patients, independently of transplantation. A meta-analysis of cross-sectional and prospective studies found an OR for cognitive impairment of 1.79 (95% CI 1.24 to 2.58) and 2.87 (95% CI 1.31 to 6.27), respectively, for CKD compared with non-CKD patients (16). The REasons for Geographic And Racial Differences in Stroke (REGARDS) study found an adjusted OR of 1.23 (95% CI 1.06 to 1.43) for cognitive impairment if CKD was present, investigating 23,400 participants (17). In the Cardiovascular Health and Cognition Study (CHCS), comprehensive neuropsychological testing yielded an adjusted hazard ratio (HR) of 1.37 (95% CI 1.06 to 1.78) for the occurrence of dementia in cases of elevated levels of creatinine (18). The risk of cognitive impairment also appears to increase progressively with declining kidney function. Tamura et al. found that the prevalence of cognitive impairment increased by 11% for each 10 mL/min/1.73 m^2^ decrease in eGFR below 60 mL/min/1.73 m^2^ (17). Seliger at al. found the adjusted HR for dementia to be 1.26 (95% CI 1.02 to 1.54) for each 88 μmol/L increase in creatinine level (18).

CKD patients often accumulate a number of risk factors associated with cognitive impairment, and this has been comprehensively reviewed by Murtaza et al. (19) and Jurgensen et al. (20). In addition to the traditional risk factors for accelerated atherosclerosis, such as diabetes, hypertension, dyslipidemia, and smoking, lifestyle factors such as low activity levels, obesity, poor diet, increased alcohol consumption, decreased engagement in mentally stimulating activities, and social disengagement play an additional role. Importantly, and more specifically, cognitive impairment in CKD has been associated with chronic inflammation, oxidative stress, hyperhomocysteinemia, uremic metabolites, anemia, and metabolic bone disease. Associated psychiatric comorbidities, such as depression and sleep disorders, must also be considered.

These cognitive hazards increase the risk of accelerated atherosclerosis, cerebral atrophy/neurodegeneration (cortical, subcortical, and hippocampal), ischemic microangiopathy, strokes, lacunes, and microbleeds and are associated with an accelerated general non-healthy aging trajectory.

Transplant-related immunosuppressive medications may have considerable neuropsychiatric side effects (21); however, comparative studies with detailed neuropsychiatric testing are scarce. Bermond et al. found an independent inverse association of glucocorticoid dose and memory function in KTRs (22). Pflugrad et al. found impaired global and visuospatial cognitive performance and that white matter hyperintensities in 85 liver transplant patients on a calcineurin inhibitor (CNI) were increased compared with those on a CNI-free immunosuppressive regimen 10 years after transplantation (23). De Marco et al. found that CNI-free immunosuppression in KTRs was associated with a decreased incidence of dementia (2). On the contrary, Taglialatela et al. found a reduced incidence of dementia in solid organ recipients taking CNI (24).

### What are the consequences?

Dementia in KTRs has been associated with graft loss and mortality. Studies within the US Renal Data System showed death-censored 3-, 5-, and 10-year graft loss rates of 11.1%, 21%, and 43.1%, respectively, in KTRs with dementia compared with 7.7%, 12.6%, and 28.8%, respectively, in KTRs without dementia. The 1-, 3-, 5-, and 10-year mortality rates were 20.8%, 46%, 64.9%, and 89.9%, respectively, in KTRs with dementia, and 7.4%, 16%, 26.3%, and 55.7%, respectively, in KTRs without dementia (2). Thomas et al. found that the 5-year graft loss rate in KTRs with dementia was 45.5% and 10.6% in those without dementia (25). However, these outcomes were only correlations; causal relations need to be investigated. However, a diagnosis of dementia in KTRs should increase care and increase awareness for disadvantageous outcomes. In addition, as vascular dementia is common among CKD patients, dysexecutive cognitive and behavioral patterns deserve particular attention. A dysexecutive syndrome manifests itself, among other things, with gait and balance impairments, apparent lack of interest, depressed mood, and physical, cognitive, and emotional inactivity putting KTPs at an increased risk of injurious falls, sarcopenia, depression, social isolation, malnutrition, poor adherence, and loss of autonomy.

### What kind of screening is appropriate?

The central element in the search for cognitive impairment outside specialized disciplines is the global cognition screening test. It is important to note that a cognitive screening test provides neither a diagnosis nor a comprehensive evaluation of cognitive performance. It is no more, but no less, than an initial assessment to objectify basic cognitive abilities and to evaluate if further workup is warranted. Furthermore, none of the existing cognitive screening tests have been validated in KTRs. Limited data exist for the MoCA (1, 3), the Dementia Detection Test (DemTect) (4), the 3MS (7, 25), the Repeatable Battery for the Assessment of Neuropsychological status (BRANS) (8), and several tests of selected cognitive domains (8, 9, 15, 26) (**Table 1**).

Based on the common association of cognitive impairment with depression, it should be ruled out before the testing of cognitive performance. Cognitive performance screening in KTRs should fulfil the following criteria of high sensitivity to capture mild cognitive impairment (MCI), ascertainment of the main characteristics of Alzheimer disease, vascular dementia, and uremic encephalopathy comprising at least the domains of memory, language, visuospatial abilities, executive functions and attention. The screening test should offer a correction for the level of education. Ideally, population-specific, region- and age-adapted normative values, and validated cutoff values should also be available.

The MoCA, DemTect, 3MS and RBANS tests meet many of these requirements. They are superior to the Mini Mental State Examination (MMSE) for the detection of mild cognitive impairment (27–30), cover all the essential cognitive domains include multiple executive function tests, and are recommended for the screening of both Alzheimer disease (11, 31–33) and vascular dementia (32, 34–36). None of these tests have been validated in KTRs or CKD patients. The MoCA has been validated in patients on hemodialysis (31), if the MMSE is applied, additional testing of executive function is essential (e.g., via clock drawing or Trail Making Test B).

It is crucial to objectify a patient’s cognitive performance via an appropriate screening test. Clinical judgment has been proven considerably inaccurate in both the general population and in KTRs (37, 38).

Alongside the central element of the screening test, additional factors can be helpful in narrowing the likelihood of dementia. Neurodegeneration and vascular disease are the main causal factors of dementia in KTRs. Therefore, the classical trajectory of its clinical presentation is a long-term and slowly progressive, or stepwise cognitive decline. From the cognitive domains of memory, language, visuospatial abilities, executive functions, and behavior, at least two need to be affected. For a diagnosis of dementia to be made, the cognitive decline needs to affect a patient’s everyday life, which is a challenging clinical judgment that is based on the individual patient’s prior performance level. All these factors need to be evaluated via consultations with both the patient and a knowledgeable informant.

Cognitive screening results should be interpreted with caution if there is considerable alteration of vital signs (e.g., very low or high blood pressure, hypoxemia, hypercapnia, fever), electrolytes, blood sugar, acid–base balance, or blood count. In addition, whether or not there is relevant interference from centrally acting medications needs to be determined. Ideally, all these factors should be excluded or corrected before cognitive performance testing. Higher-grade neuropsychiatric comorbidities, such as depression, substance use, sleep disorders, Parkinson disease, psychosis, or unfavorable/premorbid personality developments may complicate the execution of screening and the interpretation of screening results. However, it is more important that the screening actually takes place than that too much attention is paid to possible influencing factors. Professional work up can disentangle those factors.

Patients with a screening result suggesting that they have cognitive impairment should undergo professional evaluation, which is generally offered by neurologists, geriatricians, or psychiatrists (e.g. memory clinics). Depending on the individual circumstances, comprehensive professional evaluation might not always be necessary or feasible. However, the professional counseling of patients, family members and care teams is recommended.

### When should screening take place?

There is convincing evidence that cognition significantly improves after transplantation. This was found through the global cognition testing of 405 and 665 KTRs at 1 year and at a median of 1.5 (IQR 0.7, 3.4) years’ follow-up after transplantation (7, 15). It has also been found in smaller studies on the executive functions, which were carried out at 1 month (5), 3 months (6), and 1 year (8) after transplantation, and in those on memory and psychomotor speed and attention at 1 month (5), 3 months (8), and 6 months (9) after transplantation, and at 1 month (5) and 3 months (8) after transplantation, respectively.

Studies measuring cognition at admission to kidney transplantation (14, 25) assess performance under pre-dialysis or dialysis conditions. In the first days and weeks after transplantation, surgery-related burdens, high doses of centrally acting drugs, infectious complications, psychological strains, and changing levels of renal function, volume status, and electrolytes often predominate. In this context, the evaluation of cognitive functions may be confounded, or affected by delirium, which is a strong indicator of pre-existing cognitive impairment. After the immediate post-transplant phase, there is a good chance of significant cognitive improvement, as outlined above. In the long term, prognostic factors and course of cognitive abilities will decreasingly be related to the kidney transplant itself and instead to the well-known risk factors of age, CKD, genetics, and lifestyle. However, if there is suspicion of cognitive impairment, screening should not be postponed.

### Who should conduct the screening?

The screening of cognitive impairment is not limited to specialized care teams. On the contrary, as dementia in older adults is highly prevalent but often underdiagnosed, screening should be encouraged among all care team professionals (e.g., general practitioners, specialty physicians, nurses, occupational therapists, psychologists, physiotherapists). Special training in how to apply a specific general cognition screening test is therefore surely recommendable. In addition, most screening tests contain specific instructions, enabling their broad applicability.

### What are the benefits of cognitive screening?

Cognitive screening does not provide a diagnosis. However, it provides valuable information with regard to the possible deficits and risks regarding general and transplant care. Cognition screening helps to draw the attention to treatable causes of cognitive impairment, such as vitamin B_12_ (holo-transcobalamin) deficiency, thyroid disorders, sleep disturbances, depression, medication side effects, and cerebrovascular disease.

With regard to the care of KTRs with cognitive impairment, the degree of deficits is crucial. Where KTRs with mild cognitive impairment or first-stage Alzheimer disease may function independently with perfect adherence for several years, those who are at more advanced stages may need specific support to ensure graft survival and prevent complications from occurring. Severe dementia is not defined by the degree of cognitive decline but by the resulting need for institutional care (or equivalent domestic support).

Cognitive impairment may lead to non-adherence, which puts KTRs at an increased risk of rejection, graft loss, missed consultations, and other complications. Poor medical adherence has not only been associated with deficits in memory function but also with deficits in the domains of attention and execution (39). As dementia can very well present without advanced deficits in memory, the evaluation of executive abilities should always be carried out during screening. In addition, repeated de-prescribing efforts will reduce the risk of drug interactions and increase adherence to essential medications. Potentially inappropriate medications need to be used with caution; however, according to the circumstances, selected use can be valuable and very well justified.

Cognitive impairment is a strong risk factor for delirium. Hereby, comparably small additional stressors (lack of sleep, change of location, pain, infection, centrally acting medication, dysvolemia, hypoxemia, unmet physical, or mental needs) may trigger potentially hazardous delirious states. This should be anticipated when KTRs with cognitive impairment face challenges, such as hospitalizations, medical interventions, or surgery.

Dementia is frequently associated with increased vulnerability/frailty and other geriatric syndromes (e.g., falls, functional impairment, depression, incontinence, decubiti, sarcopenia, malnutrition). Therefore, it is crucial to carefully choose which diagnostic and therapeutic interventions are really necessary. The focus should be on the priorization of personal needs, the reduction of potential complications and the prevention of a disproportional “load of care”. Behavioral and psychological symptoms of dementia (BPSD) develop frequently with declining cognitive function and are often present at the time of diagnosis. Symptoms range from mild discontent to severe and challenging stubburn, aggressive, delusional, depressive, or apathetic behaviors.

In cognitive impairment, establishing a selected and reliable care team is of great value (e.g., patient, family member, general practitioner, home care, physiotherapy, nephrologist). Communication with the patient needs to be adjusted according to their level of cognitive impairment, with special attention given to medication and the selection of medical necessities to prevent harm (less is more). Dementia may place a considerable burden on family members and institutions. Therefore, the evaluation of possible caregiver burden should be an integrative part of the evaluation and care among KTRs with dementia.

In terms of the capacity for judgment in patients with cognitive impairment, it is worth keeping in mind that this can be assessed only with regard to a specific question, topic, or decision. A general ability or lack of ability for judgment does not exist. In addition, independent of the severity of cognitive impairment, there is always a partial or residual capacity for judgment that can be inferred by the patient’s communication and the caregivers’ experiences with the patient. Foresighted evaluation and documentation of patients’ thoughts, wishes, and will regarding medical and social end-of-life decisions, support appropriate future care.

Last but not least, declining cognitive function should raise our awareness of the patients’ quality of life. Attention should be placed on meeting of individual patients’ changing needs.

## Conclusion

The literature on cognitive performance in KTRs suggests a high prevalence of impaired cognition with potentially hazardous consequences. Hence, the evaluation of cognitive abilities is essential if there is a suspicion of declining cognitive performance.

As clinical judgment has been shown to be inaccurate, high-quality cognition screening tests need to be used. There are no screening tools that have been specifically validated for the KTR population. However, well-established global cognition screening tools such as the MoCA, the 3MS, or the DemTect are available and highly recommendable for KTRs. These tests meet KTR-specific requirements regarding cognitive domains included and sensitivity to mild cognitive impairment, can be conducted in less than 20 min by a minimally trained healthcare professional, offer a huge body of evidence from other populations and some experience exist in KTRs (**Table 1**). The MMSE can also be recommended provided that validated testing of executive functions is additionally carried out (e.g., clock drawing, TMT-B, verbal fluency).

With regard to an appropriate time point of testing, cognition screening should not be delayed and conducted in a timely manner if there is suspicion of impaired cognitive functions. An episode of delirium (e.g., during a KTRs’ hospital stay) indicates a pre-existing cognitive impairment until proven otherwise; therefore, special attention is needed for the follow-up of these KTRs.

Experience regarding cognitive function in KTRs is limited, patient compliance is vital, and a diagnosis of mild cognitive impairment or dementia changes management in multiple ways (as outlined above). Therefore, a professional dementia workup should be initiated if screening results are suggestive of this.

For future research and better clinical understanding, not only screening data, but also results from gold standard dementia diagnostic tools are needed. Existing cognitive screening tests need to be validated in the KTR population. This will set a base for a better understanding of cognitive impairment in KTRs regarding frequency, severity, illness trajectories, and associated factors. More data are needed regarding the cognitive performance of KTRs with regard to different age groups, education level, geographical region, comorbidities, lifestyle factors, and kidney function. This will enable the better interpretation of individual test results and sharpen the focus on high-risk constellations.

High rates of graft loss in KTRs with dementia have been attributed to declining self-care abilities and non-adherence. Future research is warranted to better understand precise causative factors and possible preventation strategies in KTRs with cognitive impairment. In the meantime, securing adherence and meeting changing needs should be a priority in KTRs with impaired cognition.

## Author contributions

SS wrote the manuscript. RK and UH-D reviewed the manuscript. All authors contributed to the article and approved the submitted version.

## References

[1] Gupta A Mahnken JD Johnson DK Thomas TS Subramaniam D Polshak T. Prevalence and correlates of cognitive impairment in kidney transplant recipients. BMC Nephrol (2017) 18(1):158. doi: 10.1186/s12882-017-0570-1 28499360 PMC5429555

[2] McAdams-DeMarco MA Bae S Chu N Gross AL Brown CH 4th Oh E. Dementia and Alzheimer's disease among older kidney transplant recipients. J Am Soc Nephrol (2017) 28(5):1575–83. doi: 10.1681/ASN.2016080816 PMC5407731 27979990

[3] Gupta A Montgomery RN Young K Mukherjee R Chakraborty S Thomas TS. Pre-transplant cognitive screening is a poor predictor of post-transplant cognitive status. Clin Transplant (2022) 36(11):e14798. doi: 10.1111/ctr.14798 35989467 PMC10691449

[4] Nöhre M Bauer-Hohmann M Klewitz F Kyaw Tha Tun EM Tegtbur U Pape L. Prevalence and correlates of cognitive impairment in kidney transplant patients using the DemTect-results of a KTx360 substudy. Front Psychiatry (2019) 10:791. doi: 10.3389/fpsyt.2019.00791 31736808 PMC6837156

[5] Harciarek M Biedunkiewicz B Lichodziejewska-Niemierko M Debska-Slizień A Rutkowski B. Cognitive performance before and after kidney transplantation: a prospective controlled study of adequately dialyzed patients with end-stage renal disease. J Int Neuropsychol Soc (2009) 15(5):684–94. doi: 10.1017/S1355617709990221 19570307

[6] Gupta A Lepping RJ Yu AS Perea RD Honea RA Johnson DK. Cognitive function and white matter changes associated with renal transplantation. Am J Nephrol (2016) 43(1):50–7. doi: 10.1159/000444334 PMC5870800 26894920

[7] Chu NM Chen X Gross AL Carlson MC Garonzik-Wang JM Norman SP. Cognitive impairment burden in older and younger adults across the kidney transplant care continuum. Clin Transplant (2021) 35(10):e14425. doi: 10.1111/ctr.14425 34272777 PMC8595550

[8] Binari LA Kiehl AL Jackson JC Feurer ID Rega SA Altuhaifi TM. Neurocognitive function changes following kidney transplant: A prospective study. Kidney Med (2022) 4(12):100560. doi: 10.1016/j.xkme.2022.100560 36507052 PMC9732409

[9] Griva K Thompson D Jayasena D Davenport A Harrison M Newman SP. Cognitive functioning pre- to post-kidney transplantation–a prospective study. Nephrol Dial Transplant (2006) 21(11):3275–82. doi: 10.1093/ndt/gfl385 16861731

[10] Teng EL Chui HC. The modified mini-mental state (3MS) examination. J Clin Psychiatry (1987) 48(8):314–8. 3611032

[11] Kalbe E Kessler J Calabrese P Smith R Passmore AP Brand M. DemTect: a new, sensitive cognitive screening test to support the diagnosis of mild cognitive impairment and early dementia. Int J Geriatr Psychiatry (2004) 19(2):136–43. doi: 10.1002/gps.1042 14758579

[12] Nasreddine ZS Phillips NA Bédirian V Charbonneau S Whitehead V Collin I. The Montreal Cognitive Assessment, MoCA: a brief screening tool for mild cognitive impairment. J Am Geriatr Soc (2005) 53(4):695–9. doi: 10.1111/j.1532-5415.2005.53221.x 15817019

[13] Bacigalupo I Mayer F Lacorte E Di Pucchio A Marzolini F Canevelli M. A systematic review and meta-analysis on the prevalence of dementia in Europe: estimates from the highest-quality studies adopting the DSM IV diagnostic criteria. J Alzheimers Dis (2018) 66(4):1471–81. doi: 10.3233/JAD-180416 PMC6294583 30412486

[14] Thind AK Rule A Goodall D Levy S Brice S Dor FJMF. Prevalence of frailty and cognitive impairment in older transplant candidates - a preview to the Kidney Transplantation in Older People (KTOP): impact of frailty on outcomes study. BMC Nephrol (2022) 23(1):283. doi: 10.1186/s12882-022-02900-w 35963988 PMC9375902

[15] Chu NM Gross AL Shaffer AA Haugen CE Norman SP Xue QL. Frailty and changes in cognitive function after kidney transplantation. J Am Soc Nephrol (2019) 30(2):336–45. doi: 10.1681/ASN.2018070726 PMC6362628 30679381

[16] Etgen T Chonchol M Förstl H Sander D. Chronic kidney disease and cognitive impairment: a systematic review and meta-analysis. Am J Nephrol (2012) 35(5):474–82. doi: 10.1159/000338135 22555151

[17] Kurella Tamura M Wadley V Yaffe K McClure LA Howard G Go R. Kidney function and cognitive impairment in US adults: the Reasons for Geographic and Racial Differences in Stroke (REGARDS) Study. Am J Kidney Dis (2008) 52(2):227–34. doi: 10.1053/j.ajkd.2008.05.004 PMC2593146 18585836

[18] Seliger SL Siscovick DS Stehman-Breen CO Gillen DL Fitzpatrick A Bleyer A. Moderate renal impairment and risk of dementia among older adults: the Cardiovascular Health Cognition Study. J Am Soc Nephrol (2004) 15(7):1904–11. doi: 10.1097/01.ASN.0000131529.60019.FA 15213280

[19] Murtaza A Dasgupta I. Chronic kidney disease and cognitive impairment. J Stroke Cerebrovasc Dis (2021) 30(9):105529. doi: 10.1016/j.jstrokecerebrovasdis.2020.105529 33323323

[20] Jurgensen A Qannus AA Gupta A. Cognitive function in kidney transplantation. Curr Transplant Rep (2020) 7:145–53. doi: 10.1007/s40472-020-00284-0 PMC7473337 32905482

[21] Zhang W Egashira N Masuda S. Recent topics on the mechanisms of immunosuppressive therapy-related neurotoxicities. Int J Mol Sci (2019) 20(13):3210. doi: 10.3390/ijms20133210 31261959 PMC6651704

[22] Bermond B Surachno S Lok A ten Berge IJ Plasmans B Kox C. Memory functions in prednisone-treated kidney transplant patients. Clin Transplant (2005) 19(4):512–7. doi: 10.1111/j.1399-0012.2005.00376.x 16008597

[23] Pflugrad H Schrader AK Tryc AB Ding X Lanfermann H Jäckel E. Longterm calcineurin inhibitor therapy and brain function in patients after liver transplantation. Liver Transpl (2018) 24(1):56–66. doi: 10.1002/lt.24984 29156491

[24] Taglialatela G Rastellini C Cicalese L. Reduced incidence of dementia in solid organ transplant patients treated with calcineurin inhibitors. J Alzheimers Dis (2015) 47(2):329–33. doi: 10.3233/JAD-150065 PMC4923720 26401556

[25] Thomas AG Ruck JM Shaffer AA Haugen CE Ying H Warsame F. Kidney transplant outcomes in recipients with cognitive impairment: a national registry and prospective cohort study. Transplantation (2019) 103(7):1504–13. doi: 10.1097/TP.0000000000002431 PMC6393218 30153224

[26] Harciarek M Biedunkiewicz B Lichodziejewska-Niemierko M Dębska-Ślizień A Rutkowski B. Continuous cognitive improvement 1 year following successful kidney transplant. Kidney Int (2011) 79(12):1353–60. doi: 10.1038/ki.2011.40 21389973

[27] Ciesielska N Sokołowski R Mazur E Podhorecka M Polak-Szabela A Kędziora-Kornatowska K. Is the Montreal Cognitive Assessment (MoCA) test better suited than the Mini-Mental State Examination (MMSE) in mild cognitive impairment (MCI) detection among people aged over 60? Meta-analysis. Psychiatr Pol (2016) 50(5):1039–52. doi: 10.12740/PP/45368 27992895

[28] Beyermann S Trippe RH Bähr AA Püllen R. Mini-mental-status-test im stationären geriatrischen bereich [mini-mental state examination in geriatrics : an evaluation of diagnostic quality. Z Gerontol Geriatr (2013) 46(8):740–7. doi: 10.1007/s00391-013-0488-6 23483351

[29] Van Patten R Britton K Tremont G. Comparing the mini-mental state examination and the modified mini-mental state examination in the detection of mild cognitive impairment in older adults. Int Psychogeriatr (2019) 31(5):693–701. doi: 10.1017/S1041610218001023 30021667

[30] Aslanzadeh F Braun S Brechbiel J Willis K Parker K Lanoye A. Re-examining popular screening measures in neuro-oncology: MMSE and RBANS. Support Care Cancer (2022) 30(10):8041–9. doi: 10.1007/s00520-022-07213-0 35771290

[31] Freitas S Simões MR Alves L Vicente M Santana I. Montreal cognitive assessment: validation study for mild cognitive impairment and Alzheimer disease. Alzheimer Dis Assoc Disord (2013) 27(1):37–43. doi: 10.1097/WAD.0b013e3182420bfe 22193353

[32] Kanser RJ VandenBussche Jantz AB Logan PM Bailey EK Kamper JE. Comparing detection of alzheimer's and vascular disease-related cognitive impairment with brief cognitive screens. J Neuropsychiatry Clin Neurosci (2022) 34(4):361–6. doi: 10.1176/appi.neuropsych.21040091 35578799

[33] Karantzoulis S Novitski J Gold M Randolph C. The Repeatable battery for the assessment of neuropsychological status (RBANS): Utility in detection and characterization of mild cognitive impairment due to Alzheimer's disease. Arch Clin Neuropsychol (2013) 28(8):837–44. doi: 10.1093/arclin/act057 23867976

[34] Freitas S Simões MR Alves L Vicente M Santana I. Montreal Cognitive Assessment (MoCA): validation study for vascular dementia. J Int Neuropsychol Soc (2012) 18(6):1031–40. doi: 10.1017/S135561771200077X 22676901

[35] Calabrese P Sitek EJ Korczyn AD Dong Y Manso-Calderón R Sierra-Beltrán M. The assessment of cognitive and behavioural disturbances in vascular cognitive impairment (VCI) - recommendations of an expert working group. Neurol Neurochir Pol (2021) 55(4):333–45. doi: 10.5603/PJNNS.a2021.0035 34096014

[36] Zhang CY Deng YM Li DM Shi QY Yang XP Zhao YH. Reliability and validity of the repeatable battery for assessment of neuropsychological status scale in evaluation of vascular cognitive impairment in elderly han population. Noro Psikiyatr Ars (2022) 59(2):147–50. doi: 10.29399/npa.27854 PMC9142023 35685057

[37] Chodosh J Petitti DB Elliott M Hays RD Crooks VC Reuben DB. Physician recognition of cognitive impairment: evaluating the need for improvement. J Am Geriatr Soc (2004) 52(7):1051–9. doi: 10.1111/j.1532-5415.2004.52301.x 15209641

[38] Gupta A Thomas TS Klein JA Montgomery RN Mahnken JD Johnson DK. Discrepancies between perceived and measured cognition in kidney transplant recipients: implications for clinical management. Nephron (2018) 138(1):22–8. doi: 10.1159/000481182 PMC5828957 29049997

[39] Stilley CS Bender CM Dunbar-Jacob J Sereika S Ryan CM. The impact of cognitive function on medication management: three studies. Health Psychol (2010) 29(1):50–5. doi: 10.1037/a0016940 PMC2807986 20063935

